# Extracellular matrix components and culture regimen selectively regulate cartilage formation by self-assembling human mesenchymal stem cells in vitro and in vivo

**DOI:** 10.1186/s13287-016-0447-4

**Published:** 2016-12-09

**Authors:** Johnathan Ng, Yiyong Wei, Bin Zhou, Aonnicha Burapachaisri, Edward Guo, Gordana Vunjak-Novakovic

**Affiliations:** 1 Department of Biomedical Engineering, Columbia University, 622 West 168th Street, VC12-234, New York, NY 10032 USA; 2 Department of Medicine, Columbia University, New York, NY USA; 3 Columbia University, 345 Engineering Terrace, 1210 Amsterdam Avenue, New York, NY 10027 USA

## Abstract

**Background:**

Cartilage formation from self-assembling mesenchymal stem cells (MSCs) in vitro recapitulate important cellular events during mesenchymal condensation that precedes native cartilage development. The goal of this study was to investigate the effects of cartilaginous extracellular matrix (ECM) components and culture regimen on cartilage formation by self-assembling human MSCs in vitro and in vivo.

**Methods:**

Human bone marrow-derived MSCs (hMSCs) were seeded and compacted in 6.5-mm-diameter transwell inserts with coated (type I, type II collagen) or uncoated (vehicle) membranes, at different densities (0.5 × 10^6^, 1.0 × 10^6^, 1.5 × 10^6^ per insert). Pellets were formed by aggregating hMSCs (0.25 × 10^6^) in round-bottomed wells. All tissues were cultured for up to 6 weeks for in vitro analyses. Discs (cultured for 6, 8 or 10 weeks) and pellets (cultured for 10 weeks) were implanted subcutaneously in immunocompromised mice to evaluate the cartilage stability in vivo.

**Results:**

Type I and type II collagen coatings enabled cartilage disc formation from self-assembling hMSCs. Without ECM coating, hMSCs formed dome-shaped tissues resembling the pellets. Type I collagen, expressed in the prechondrogenic mesenchyme, improved early chondrogenesis versus type II collagen. High seeding density improved cartilage tissue properties but resulted in a lower yield of disc formation. Discs and pellets exhibited compositional and organizational differences in vitro and in vivo. Prolonged chondrogenic induction of the discs in vitro expedited endochondral ossification in vivo.

**Conclusions:**

The outcomes of cartilage tissues formed from self-assembling MSCs in vitro and in vivo can be modulated by the control of culture parameters. These insights could motivate new directions for engineering cartilage and bone via a cartilage template from self-assembling MSCs.

## Background

The articular cartilage has a limited ability to regenerate upon injury. Current approaches to treat focal cartilage lesions include autograft, mosaicplasty, and autologous chondrocyte implantation [1, 2]. However, these methods are limited by donor site morbidity and other complications. Thus, there is an ongoing effort toward developing stem cell-based therapies [3].

In cartilage tissue engineering (TE), scaffolding methods used successfully with chondrocytes have resulted in subnormal cartilage formation by mesenchymal stem cells (MSCs) – the most attractive cell source for clinical application [4]. In contrast, self-assembly methods recapitulated mesenchymal condensation during joint development, and enhanced the in vitro chondrogenesis of MSCs [5–8].

In pellet culture, cells form spherical aggregates that deposit matrix and grow over time [5, 6]. In disc culture, multiple layers of cells deposit matrix and form a disc that grows in thickness over time [8, 9]. Cartilage formed in pellet culture of self-assembling MSCs lacks physiological stratification and is not well suited for cartilage repair due to its tendency to undergo endochondral ossification in vivo [3, 10, 11]. While the pellet culture has been well established, the exact conditions for cartilage disc culture are not known [8, 9, 11–23]. In some cases, MSCs could not form discs due to the forces associated with self-assembly (Table 1). Furthermore, it remains to be determined how the conditions of MSC self-assembly in vitro affect cartilage fate in vivo.

**Table 1:** Summary of studies on disc culture of self-assembling chondrocytes or MSCs for in vitro cartilage formation

Cell type	Source	Density (× 10^6^/mm^2^)	Insert type	Substrate	Disc formation (Yes/No)	Thickness (mm)	Reference
Chondrocytes	Bovine MCC joint	0.050	Millicell PTFE	Type II collagen	Yes	-	[9]
	Bovine MCC joint	0.016	Millicell PTFE	Type II collagen	Yes	0.3	[12]
	Bovine MCC joint	0.160	-	CPP	Yes	0.94	[13]
	Sheep MCC joint	0.160	-	CPP	Yes	0.5	[14]
	Bovine hock joint	0.067	Millicell PTFE	Type II collagen	Yes	0.45	[15]
	Bovine MCC joint	0.033	Millicell PTFE	Type II collagen	Yes	-	[16]
	Bovine distal femur	0.172	-	Agarose	Yes	0.15	[17]
	Bovine distal femur	0.289	-	Agarose	Yes	0.8	[18]
	Human femoral cartilage	0.006	Millicell PTFE	Type I/Type II collagen	Yes	0.15	[19]
	Human articular cartilage	0.102	-	Agarose	Yes	0.6	[20]
MSCs	Human BM	0.015	Corning PC	None	Yes	0.8	[8]
	Porcine BM	0.392	Millicell PET	None	Yes	1.3	[21]
	Human BM	0.015	Corning PC	None	No	-	[11]
	Sheep BM	0.033	Millicell PTFE	Type IV collagen/CPP	Yes	0.5	[22]
	Equine cord blood	0.033	Millicell	Fibronectin	Yes	0.1	[23]
	Human BM	0.102	-	Agarose	No	-	[20]

In some cases, MSCs failed to form discs

*MSC* mesenchymal stem cell, *MCC* convex condyle of the metacarpus, *CPP* calcium polyphosphate, *BM* bone marrow

We hypothesized that (i) the membrane extracellular matrix (ECM) coating and (ii) the initial cell seeding density determine functional disc formation in vitro. To this end, we investigated if the control of these two parameters would enable reliable formation of cartilage discs by self-assembling MSCs. Further, we asked whether the in vivo fate of cartilage can be modulated by the self-assembly regimen (disc versus pellet) and the length of in vitro culture.

To test the hypotheses, we analyzed gene expression, biochemical, mechanical, and morphological changes of self-assembling human MSCs (hMSCs) in membrane inserts with or without ECM coating, following chondrogenic induction at different cell seeding densities. Further, we compared the composition and organization of the discs and pellets in vitro and in vivo. Finally, we investigated the in vivo fate of discs cultured under different durations in vitro. Overall, the study was designed to clarify the effects of in vitro culture parameters on the in vitro properties and in vivo fate of cartilage formed by self-assembling hMSCs.

## Methods

### Cell source and preparation

Fresh bone marrow aspirates were obtained from Cambrex (East Rutherford, NJ, USA) and processed as in our previous studies. To compare multiple treatment groups in vitro and in vivo, we used an aspirate that has been well characterized and shown to undergo robust differentiation [7, 24]. Bone marrow-derived hMSCs were isolated and expanded in high-glucose Dulbecco’s modified Eagle’s medium (DMEM) containing 10% fetal bovine serum, 1% Pen-Strep and 1 ng/mL of fibroblast growth factor-2 (FGF-2). At the end of the fourth passage, hMSCs were seeded for experiments. Several groups have reported the use of hMSCs at the end of the fourth passage for chondrogenic differentiation previously [4, 25]. All reagents were from Life Technologies (Carlsbad, CA, USA), unless specified otherwise.

### Cell seeding and differentiation

For chondrogenic differentiation, serum-free chondrogenic media consisting of high-glucose DMEM, ITS+ supplement (BD Biosciences, San Jose, CA, USA), 5 mM L-proline, HEPES, sodium pyruvate, dexamethasone, 50 μM ascorbic acid (Sigma-Aldrich, St. Louis, MO, USA) and 10 ng/mL transforming growth factor beta-3 (TGF-β3) (PeproTech, Rocky Hill, NJ, USA) was used. All reagents were from Life Technologies, unless otherwise specified.

For disc formation, 6.5-mm-diameter transwell inserts with polycarbonate membranes were used (Corning, Corning, NY, USA). To each transwell insert, 50 μL of vehicle (1 mM acetic acid with ethanol), 1.5 mg/mL type I collagen (BD Biosciences, San Jose, CA, USA) or type II collagen (Elastin Products, Owensville, MI, USA) was added and the transwell inserts were air dried overnight. The coated transwell inserts were rinsed briefly with phosphate-buffered saline (PBS) and hMSCs were seeded at various densities (0.5 × 10^6^, 1 × 10^6^, or 1.5 × 10^6^ cells per well). After which, the 24-well plates loaded with transwells were centrifuged at 200 g for 5 minutes. The next day, chondrogenic media was added so that every transwell was fully submerged. Medium was changed every 2 days for up to 6 weeks. For implantation studies, discs were cultured for 6 weeks, 8 weeks, and 10 weeks prior to implantation.

For pellet formation, 0.25 × 10^6^ hMSCs were seeded in a low-attachment round-bottomed 96-well plate (Thermo Fisher Scientific, Waltham, MA, USA), and the plates were centrifuged at 200 g for 5 minutes. The cell density was selected based on our previous study, where pellet formation was evaluated at different seeding densities, 0.25 × 10^6^ was optimal for the formation of stable pellets [7]. Medium was changed twice a week for up to 6 weeks. For implantation studies, pellets were cultured for 10 weeks prior to implantation.

### Subcutaneous implantation in severe combined immunodeficient (SCID) mice

All animal experiments followed federal guidelines and were conducted under a protocol approved by the Columbia University Animal Care and Use Committee. In vitro cultured tissues were implanted into subcutaneous pouches of 8–10-week-old female SCID mice (Jackson Laboratory, Bar Harbor, ME, USA). For each animal, two incisions were made, and one tissue was implanted into each subcutaneous pocket. For consistency, we used cells from one bone marrow aspirate that has been extensively characterized in several previous studies. The micro CT imaging (μCT) and histological evaluation were done using *n* = 4 per group and time points (6, 8, and 10 weeks). After implantation, the skin was closed with two sutures, and mice were monitored daily. No signs of discomfort were observed following surgery in any of the animals throughout the study. Endochondral ossification was evident after 4 weeks of implantation both in discs and pellets that were cultured for 10 weeks. Thus, we determined that 4 weeks was the shortest duration of implantation for evaluating endochondral ossification. Samples were explanted at 4 weeks and analyzed.

### Gene expression analysis

Samples (*n* = 4 per group) were crushed with pestles and homogenized by mixing in the TRIzol reagent. RNA was extracted according to the manufacturer’s instructions using the TRIzol method. The quantity of RNA was measured on the Nanodrop ND1000 (Thermo Fisher Scientific). Following treatment with DNase I removal kit, cDNA synthesis was performed using the Applied Biosystems (Waltham, MA, USA) High Capacity kit according to the manufacturer’s instructions. Quantitative RT-PCR analysis was done using 20 ng cDNA per reaction and the Applied Biosystems SYBR® Green PCR Master Mix. The expression of target genes was normalized to GAPDH, unless otherwise stated, and the mean cycle threshold (CT) values of technical duplicates were used for all calculations. The values indicated for all target genes (2^-∆Ct^) are “fold expression relative to GAPDH”, unless otherwise stated. Data for the same gene were compared among all groups at different time points. All reagents were from Life Technologies. All primers (Table 2) were synthesized by Life Technologies.

**Table 2:** List of primers used for qPCR

Gene	Forward sequence	Reverse sequence
ACAN	CCCCTGCTATTTCATCGACCC	GACACACGGCTCCACTTGAT
C4ST	CATCTACTGCTACGTGCCCA	CTTCAGGTAGCTGCCCACTC
C6ST	CTCGGAGCAGTTCGAGAAGTG	CGCCAGTTTGTAGCCGAAGA
CHM1	CTGGATCACGAAGGAATCTGT	ACCATGCCCAAGATACGGG
COL10A1	CATAAAAGGCCCACTACCCAAC	ACCTTGCTCTCCTCTTACTGC
COL1A1	GATCTGCGTCTGCGACAAC	GGCAGTTCTTGGTCTCGTCA
COL2A1	AGACTTGCGTCTACCCCAATC	GCAGGCGTAGGAAGGTCATC
GAPDH	TGTTGCCATCAATGACCCCTT	CTCCACGACGTACTCAGCG
MATN3	TCTCCCGGATAATCGACACTC	CAAGGGTGTGATTCGACCCA
RUNX2	CCGTCTTCACAAATCCTCCCC	CCCGAGGTCCATCTACTGTAAC
SOX9	AGCGAACGCACATCAAGAC	CTGTAGGCGATCTGTTGGGG

### Biochemical analysis

DNA, sulfated glycosaminoglycan (GAG), and collagen (COL) contents were measured as previously described (2). All reagents unless otherwise specified were from Sigma-Aldrich. Briefly, the samples (*n* = 4) were blotted dry and weighed. Subsequently, the samples were digested in 0.5 mL proteinase K solution at 60 °C. With the sample digests, the DNA content was determined using the Molecular Probes Picogreen assay (Life Technologies). The GAG content of the sample digests was determined using the 1,9-dimethylmethylene blue (DMMB) dye calorimetric assay with chondroitin-6-sulfate as a standard. Acid hydrolysis of the sample digests was used to determine hydroxyproline content, assumed to be 10% of the total collagen content.

### Mechanical testing

The compressive Young’s modulus of the cartilage was measured on samples submerged in PBS using unconfined compression, by a previously described method [7]. From the tissue samples (*n* = 4 per group), 3-mm-diameter discs were cored using a biopsy punch. The disc thickness was measured by the position of tissue contact to that of the platform. The cylindrical constructs were compressed at a rate of 100 nm/s (approximately 0.02% strain per second) for up to 2000 s, and the compressive load was measured. Young’s modulus was calculated from the linear slope (20–30% strain) of the stress–strain curve. Full-thickness cartilage discs were obtained from the tibial plateau of 2-month-old calves (Green Village Packing, Green Village, NJ, USA) to serve as a reference.

### Histology and immunohistochemistry

For histology, samples were fixed in 10% formalin for 24 hours, decalcified with Immunocal (Thermo Fisher Scientific), dehydrated in ethanol, embedded in paraffin, and sectioned to 5 μm. Sections were stained for (i) hematoxylin and eosin (H&E), (ii) Alcian Blue with Nuclear Fast Red, (iii) Picosirius Red, and (iv) Movat’s pentachrome. The use of Movat’s pentachrome to evaluate cartilage/bone after implantation is widely reported. The green color indicates the presence of both collagens (staining yellow) and glycosaminoglycans (staining blue by Alcian Blue, which is one of the components of Movat’s stain). All reagents were from Sigma-Aldrich, unless otherwise specified. Immunohistochemistry was performed using the following antibodies: type I collagen, type II collagen, type X collagen, lubricin, and osteopontin (Abcam, Cambridge, MA, USA). Sections were processed according to manufacturer’s instructions. Immunobinding was detected with biotinylated secondary antibodies using the Vectastain ABC kit (Vector Laboratories, Burlingame, CA, USA). Images were acquired using an Olympus FSX-100 microscope (Olympus, Center Valley, PA, USA).

### Micro CT imaging (μCT) and standard morphological analysis

For μCT, samples were fixed in 10% formalin for 24 hours at room temperature, rinsed briefly, and kept in PBS. Three-dimensional high-resolution images were obtained for each sample using a μCT system (VivaCT 40; Scanco Medical AG, Bassersdorf, Switzerland). The grayscale images were binarized using a global threshold. Standard morphological parameters such as mineral volume (BV) and mineral density (MD) were evaluated for each sample using the standard morphological analysis software on the VivaCT 40 system.

### Statistical analysis

All quantitative results are presented as mean ± SEM (*n* = 4 per data point). Statistical analysis was performed with Prism (GraphPad, San Diego, CA, USA), using the Student *t* test or one-way ANOVA with Tukey’s post hoc test. Significant differences are denoted as ^*^(*p* < 0.05), ^**^(*p* < 0.01) and ^***^(*p* < 0.001).

## Results

### Morphological and histological analysis of cartilage formed by self-assembling hMSCs

**Fig. 1:**
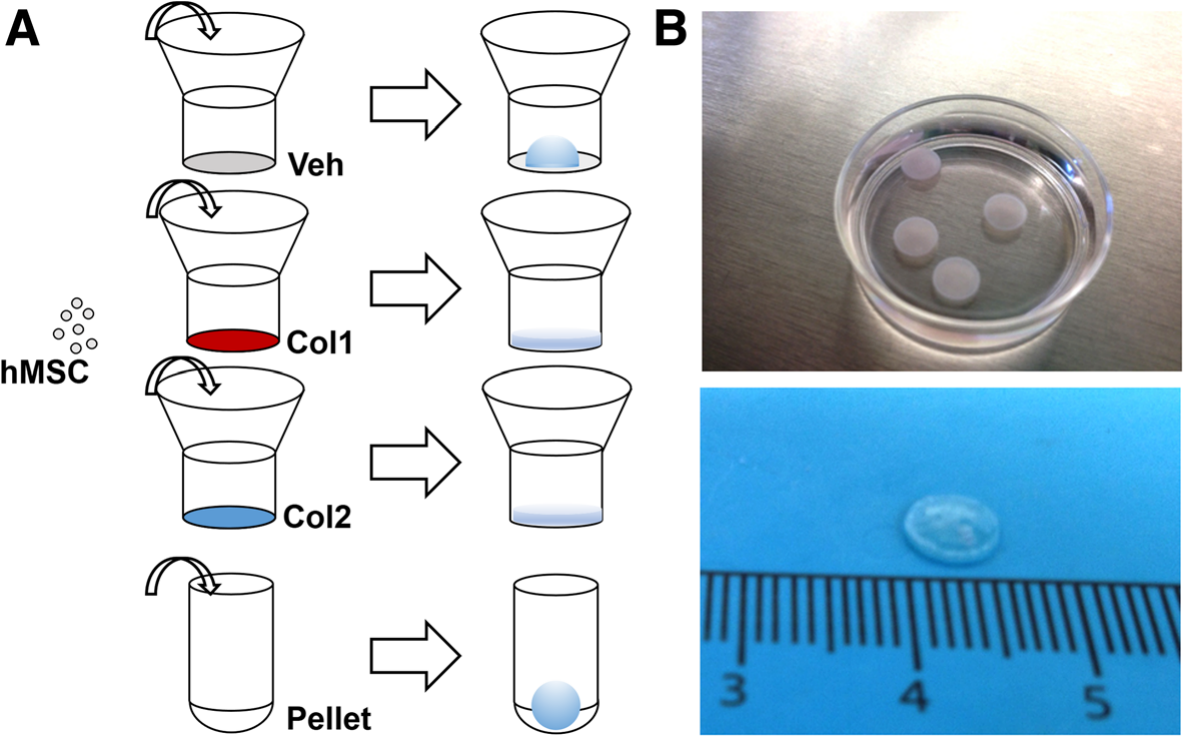
Self-assembly methods for cartilage formation by human mesenchymal stem cells (hMSCs). **a** *Top*, disc culture: hMSCs were seeded in transwell inserts coated with vehicle control (*Veh*), type I collagen (*Col1*), or type II collagen (*Col2*); Veh group failed to form discs. *Bottom*, pellet culture: hMSCs were seeded in round-bottom wells and formed spherical aggregates. **b** Representative images of hyaline discs formed in coated transwells are shown

**Fig. 2:**
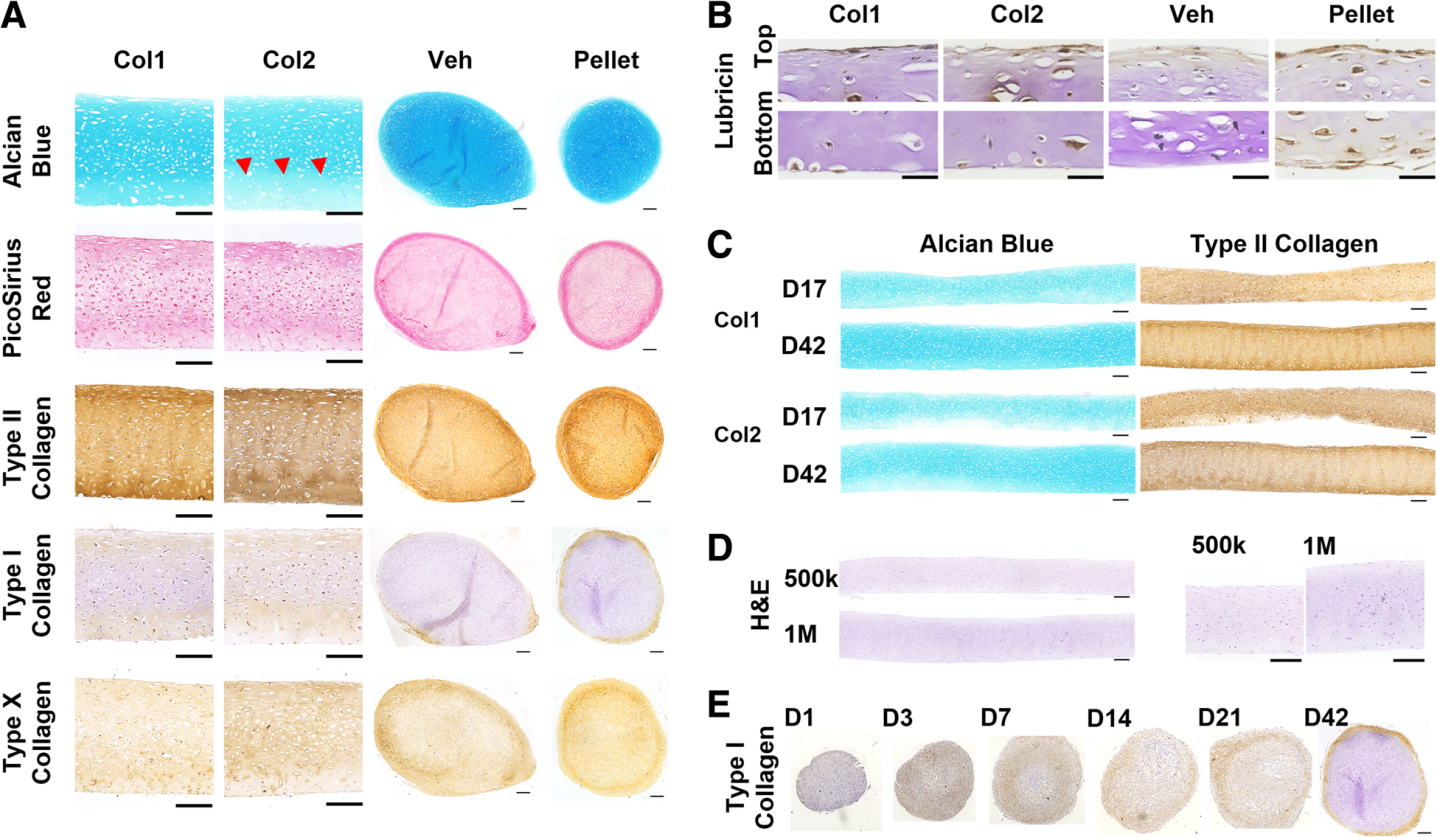
Representative histological stains. **a** Detailed view of type I collagen (*Col1*) and type II collagen (*Col2*) discs at day 42, compared with whole tissue view for the vehicle control (*Veh*) and pellet. Poor ECM deposition in the deep zone was seen in Col2 discs (*arrowheads*). Vehicle control condensed into a hemisphere. Type I collagen-rich fibrillar structure formed at the surface of the pellet and the rounded surface of the vehicle control. Discs exhibited more uniform type II collagen and GAG distribution. GAG was visibly denser at the center of the pellet. Scale bar: 200 μm. **b** Immunohistochemical stains found lubricin on the entire pellet surface but only on the top surface of the discs. Scale bar: 50 μm. **c** Whole tissue comparisons of Col1 and Col2 discs after 17 and 42 days of chondrogenic induction. Both groups formed stratified discs that grew in thickness over time. Less ECM was observed in the deep zone of Col2 discs. Scale bar: 200 μm. **d** Hematoxylin and eosin (*H&E*) stains of Col1 discs seeded at 0.5 × 10^6^ and 1.0 × 10^6^. Scale bar: 200 μm. **e** Immunohistochemical type I collagen stains of pellets at different time points after chondrogenic induction found initial expression throughout the pellet, and peripheralization over time. Scale bar: 200 μm

Type I collagen and type II collagen are important fibrillar collagens that are expressed during different stages of chondrogenesis [26, 27]. Multiple layers of hMSCs were seeded on transwell membrane inserts coated with type I collagen (Col1), type II collagen (Col2), or vehicle (Veh), and allowed to self-assemble. Pellets were also formed from self-assembling hMSCs in round-bottomed wells (Fig. 1a). Following chondrogenic induction, the Col1 and Col2 groups formed 6.5 mm hyaline discs, but not the Veh group (Fig. 1b). The lack of ECM coating in the Veh group abrogated disc formation and resulted in condensation, yielding a hemispherical tissue that resembled the pellet (Fig. 2a). The Veh hemispheres and pellets exhibited rich sulfated glycosaminoglycan (sGAG) deposition (shown by Alcian Blue) in the center regions, and dense fibrillar structures (shown by PicoSirius Red) that were rich in type I collagen at the surface of the tissues. In contrast, the Col1 and Col2 discs were stratified and exhibited more homogenous deposition of sGAG and type II collagen. To highlight the organizational differences, the tissues were stained for lubricin, the superficial zone protein. Whereas lubricin lined the entire surface of the pellets, its expression on the discs was limited to the top surface but not the bottom side in contact with the membrane. Although resembling the pellets, the Veh hemispheres was lined with lubricin only at the surface but not the bottom side in contact with the membrane (Fig. 2b).

Whole tissue comparison between the Col1 and Col2 discs revealed an overall increase in the deposition of sGAG and type II collagen with prolonged chondrogenic induction, from day 17 to day 42. However, the deposition of ECM rich in sGAG and type II collagen was only observed near membrane coated with Col1, not Col2. This was evident at both time points evaluated following chondrogenic induction, with a partial recovery of ECM deposition in the Col2 discs seen at day 42 (Fig. 2c). Using Col1 as the choice coating, the effects of cell seeding density on tissue morphology was evaluated. Increasing cell seeding density from 0.5 × 10^6^ to 1 × 10^6^ per well increased the disc thickness without causing appreciable changes in tissue morphology (Fig. 2d).

To investigate the prevalence of type I collagen during chondrogenesis of self-assembling hMSCs, pellets were stained for type I collagen at different time points following chondrogenic induction. Type I collagen was expressed in early condensed mesenchymal bodies, and reduced to the perichondral margin over time (Fig. 2e). This confirms previous findings highlighting the importance of type I collagen for early chondrogenesis during mesenchymal condensation.

### Compressive and physical properties of cartilage formed by self-assembling hMSCs

**Fig. 3:**
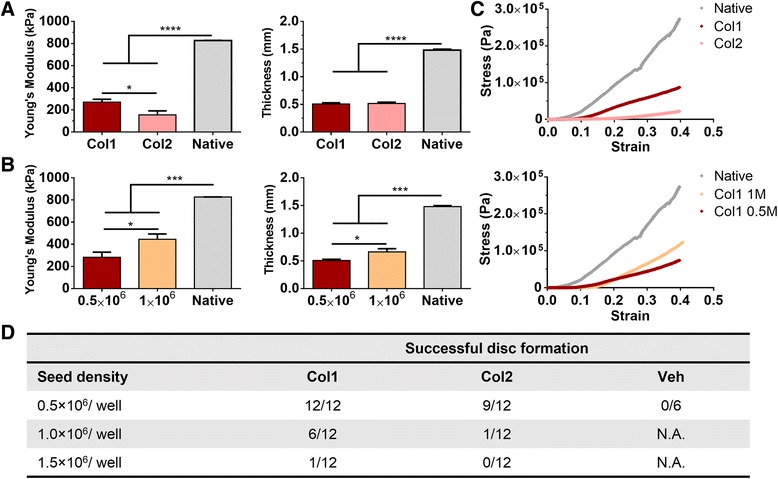
Mechanical and physical properties of discs formed by self-assembling hMSCs. **a** Young’s modulus of engineered cartilage discs at day 42 measured by unconfined compressive loading. Type I collagen (*Col1*) discs outperformed type II collagen (*Col2*) discs. Increasing seeding density from 0.5 × 10^6^ to 1.0 × 10^6^ further improved Col1 discs. Only successfully formed discs were evaluated. **b** Disc thicknesses at day 42. Col1 and Col2 discs exhibited similar thicknesses; increasing seeding density increased the thickness of Col1 discs. **c** Representative stress–strain curves. **d** Comparison of the frequency of successful disc formation. Col1 coating enabled the highest yield of disc formation, but this effect was abolished at high seeding density (1.5 × 10^6^ per well). *Veh* vehicle

In agreement with the results of histological analysis, mechanical evaluation revealed that the compressive modulus of Col1 discs (approximately 280 kPa) was significantly higher than that of the Col2 discs (approximately 170 kPa), with little difference in tissue thickness. However, neither group reached the level of native cartilage (approximately 800 kPa) (Fig. 3a). Consistent with our previous findings, cartilage formed by self-assembling hMSCs achieved a compressive modulus exceeding that of cartilage formed in scaffold culture by MSCs reported in earlier studies [7]. Increasing the cell seeding density from 0.5 × 10^6^ per well (0.015 × 10^6^/cm^2^) to 1 × 10^6^ per well (0.030 × 10^6^/cm^2^) tended to increase the thickness (from 0.5 mm to 0.7 mm) and the compressive modulus (from 280 kPa to 400 kPa) of Col1 discs (Fig. 3b). Comparing the stress–strain curves, the engineered and native tissues exhibited similar profiles with toe and elastic regions. However, the toe region (0–10% strain) associated with the native tissue was smaller than that of the engineered tissue (0–15% strain). Also, yield points and subsequent escalations were seen in the native tissues but not the engineered tissues, which remained elastic through 40% strain (Fig. 3c). These differences could be attributed to the maturity of the native cartilage (adult bovine) used as a reference.

In addition to the compressive modulus and thickness, the frequency of successful disc formation was assessed. At 0.5 × 10^6^ per well, the Col1 group formed disc in all cases (100%; 12/12) but not the Col2 group (75%; 9/12). The Veh group failed to form discs in all cases. Increasing cell seeding density to 1 × 10^6^ per well resulted in less frequent disc formation in the Col1 group (50%; 6/12) and almost no disc formation (8%; 1/12) in the Col2 group (Fig. 3d). Further increase to 1.5 × 10^6^ per well almost abolished disc formation (1 out of 12) in the Col1 group. Thus, Col1 enables more reliable formation of cartilage discs with better mechanical properties, but only up to a critical seeding density.

### Biochemical content of cartilage formed by self-assembling hMSCs

**Fig. 4:**
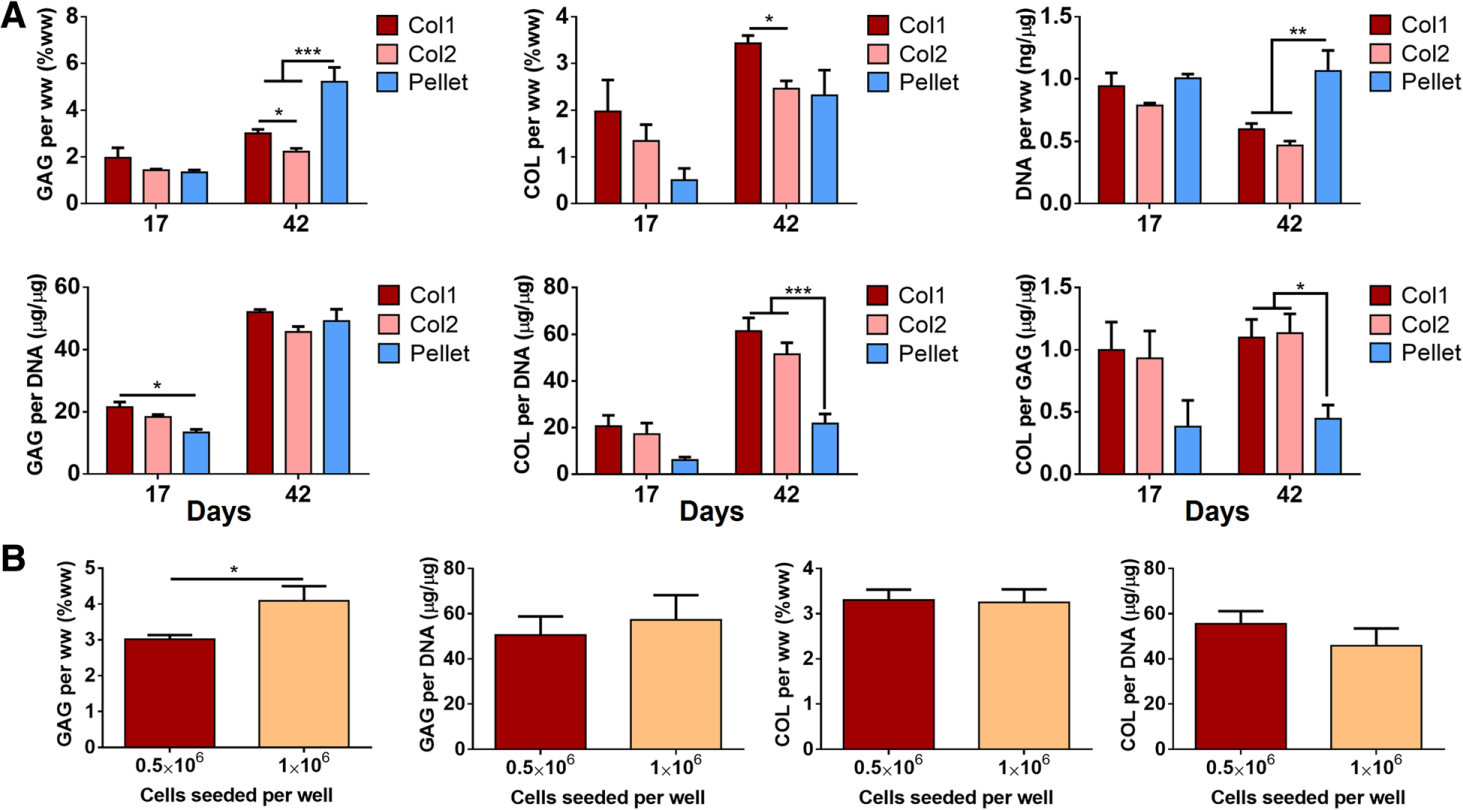
Biochemical quantification of glycosaminoglycan (*GAG*), collagen (*COL*) and cellular (*DNA*) contents. **a** Comparisons of type I collagen (*Col1*) discs, type II collagen (*Col2*) discs and pellets after 17 days or 42 days of chondrogenic induction. At day 42, pellets exhibited higher GAG and DNA contents (per wet weight) and discs exhibited higher COL/DNA and COL/GAG ratios; Col1 discs had higher GAG and DNA contents than Col2 discs. **b** Comparisons of Col1 discs at different seeding densities. Doubling seeding density from 0.5 × 10^6^ to 1.0 × 10^6^ increased GAG content (per wet weight) but not COL content. Only successfully formed discs were evaluated in the Col1 and Col2 groups

In agreement with the results of histological and mechanical evaluations, biochemical analysis revealed compositional differences between the Col1 and Col2 groups. Both the sGAG and collagen (COL) contents, normalized to wet weight, were higher in the Col1 group than the Col2 group and the differences reached significance at day 42. Comparing the pellets with the discs, compositional differences in agreement with histological analysis were also identified. Whereas the pellets had a higher sGAG content, the discs had a higher COL content, and consequently a higher COL to sGAG ratio. The higher COL content can be attributed to increased collagen productivity of cells differentiated in the disc culture than in pellet culture, as indicated by COL content normalized to DNA content (Fig. 4a).

Increasing the cell seeding density from 0.5 × 10^6^ to 1 × 10^6^ per well resulted in an increase in the sGAG content (normalized to wet weight) of the Col1 group, but not the COL content. Normalizing to DNA content, the similar sGAG/DNA ratios suggest that the increase in sGAG content could be due to greater cellularity (Fig. 4b).

### Gene expression analysis of cartilage formed by self-assembling hMSCs

**Fig. 5:**
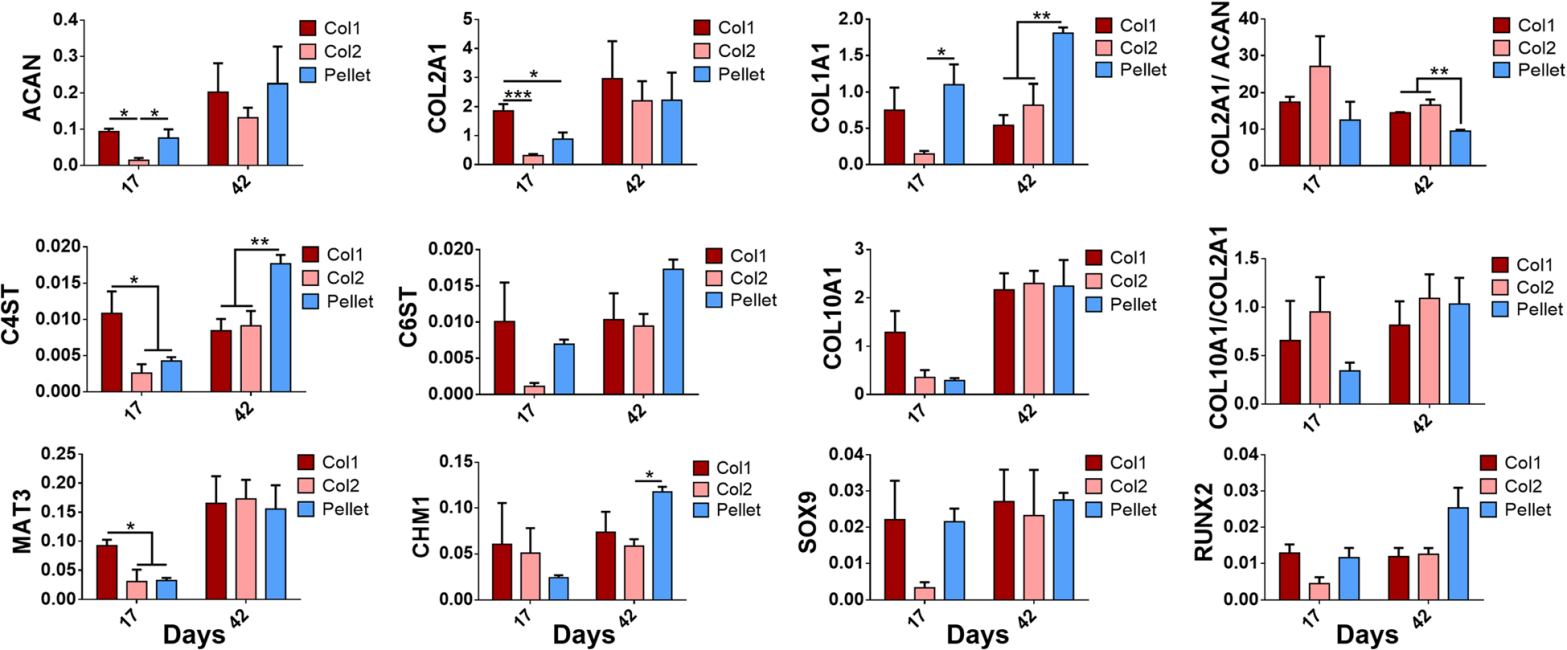
Gene expression analysis. Data are shown for type I collagen (*Col1*) discs, type II collagen (*Col2*) discs and pellets after 17 or 42 days of chondrogenic induction (by quantitative real-time polymerase chain reaction, qPCR). Col2 discs displayed lower expression of chondrogenic markers at day 17 than Col1 discs. Pellets displayed lower COL2A1/ACAN ratio and higher expression of COL1A1 than discs at day 42. All gene expression values expressed as 2^-∆Ct^ (normalized to GAPDH unless otherwise stated), and compared among all groups at different time points. Only successfully formed discs were evaluated in the Col1 and Col2 groups

To further investigate differences identified by histological, mechanical and biochemical analyses, the gene expression of important cartilage markers were analyzed. In agreement with results at the phenotypic levels, the expression of important cartilage markers (ACAN, COL2A1, C4ST, MAT3) was significantly higher in the Col1 group than the Col2 group after 17 days of chondrogenic induction, but not after 42 days (Fig. 5). The early deficits in gene expression indicate that Col2 coating delayed the onset of chondrogenesis by self-assembling hMSCs and resulted in poor long-term ECM deposition and mechanical properties.

Gene expression analysis also confirmed a cellular basis for compositional differences between the discs and the pellets as the ratio of COL2A1 to ACAN expression by cells differentiated in disc culture was higher than that in pellet culture. Greater expressions of chondroitin sulfotransferases (C4ST and C6ST) by cells in pellet culture also suggest increased GAG sulfation, which might have contributed to the higher sGAG content. Furthermore, COL1A1 expression was significantly higher in the pellets than in the discs, confirming the results of histological analysis, which revealed dense fibrillar structures rich in type I collagen at the surface of the pellets. However, COL10A1 expression was similar among the different groups. This suggests that disc culture does not prevent chondrocyte hypertrophy, despite promoting hyaline cartilage formation (Fig. 5).

### In vivo outcomes of ectopically implanted cartilage formed by self-assembling hMSCs

**Fig. 6:**
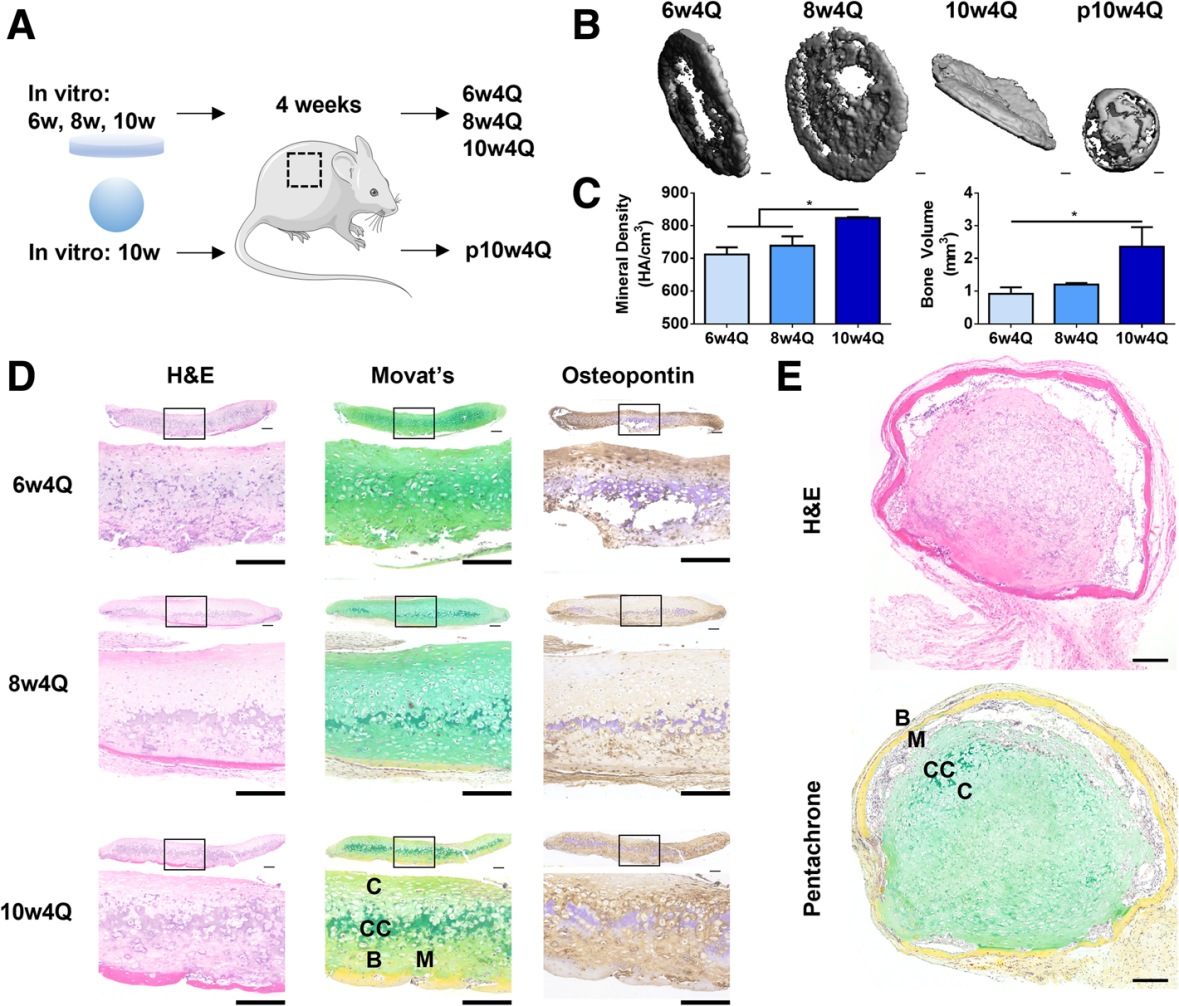
Evaluation of implantation outcomes by histological and μCT analysis. **a** Discs cultured for 6 weeks, 8 weeks or 10 weeks, as well as pellets cultured for 10 weeks were implanted subcutaneously in mice. After 4 weeks, tissues were explanted for analysis. **b** Representative μCT images show that mineralization of discs increased with length of culture, and was associated with an early mineralization at the rim. Whereas the discs formed a sheet of mineral, the pellet formed a spherical shell. **c** Mineral density and volume increased with the length of culture; 10w4Q discs exhibited higher mineral density and volume than 6w4Q and 8w4Q discs. **d** Whole tissue and detailed histological stains of discs after endochondral ossification. Scale bar: 200 μm. Discs were more mineralized with length of culture and mature bone was seen in 10w4Q discs. Bone formed at the bottom of the discs and the infiltrating marrow-like cells were observed. Non-calcified cartilage remained at the surface. **e** Whole tissue histological stains of pellets after endochondral ossification. Scale bar: 200 μm. Bone formed at the surface, with infiltrating marrow-like cells beneath gradually resorbing the calcified cartilage. *H&E* hematoxylin and eosin

Next, we investigated the effects of the culture regimen on the in vivo fate of the cartilage formed by self-assembling hMSCs in vitro. We implanted pellets, as well as discs cultured for different durations in vitro, subcutaneously in SCID mice (Fig. 6a). After 4 weeks in vivo, μCT and histological analysis of the explanted tissues revealed interesting morphological and compositional differences. As expected, the pellets underwent endochondral ossification and μCT documented the formation of a spherical shell of mineralized tissue. Movat’s pentachrome revealed an outer layer of mature bone, under which marrow-like cells infiltrated into the extensively calcified cartilage interior (Fig. 6b, e). The discs also underwent endochondral ossification but exhibited a remarkably different tissue organization (Fig. 6b, d). Mature bone and infiltrating marrow-like cells lined the bottom of the disc, and the calcified cartilage was directly above the nascent bone. Residual cartilage was observed at the superficial zone.

Furthermore, the rate of endochondral ossification in the discs increased with the duration of in vitro chondrogenic induction. After 4 weeks in vivo, discs cultured for 10 weeks (10w4Q) exhibited greater mineral volume and density than discs cultured for 6 weeks or 8 weeks (6w4Q, 8w4Q) (Fig. 6c). Movat’s pentachrome revealed distinct mature bone formation only in the 10w4Q discs (Fig. 6d). Interestingly, 6w4Q discs were mineralized prevalently at the rim in contrast to the 8w4Q discs and the 10w4Q discs that were progressively more mineralized in the center (Fig. 6b). Collectively, the results show that the culture regimen in vitro modulates endochondral ossification of cartilage formed by self-assembling hMSCs in vivo.

## Discussion

The requirements for cartilage disc formation by self-assembling MSCs are not well understood (Table 1). We showed that the ECM coating and seeding density are important determinants of functional cartilage disc formation by self-assembling hMSCs. Type I collagen enhanced chondrogenesis and promoted disc formation, whereas high cell density improved tissue properties but resulted in less frequent disc formation. Comparing pellet and disc cultures, we identified compositional and morphological differences during in vitro culture and following in vivo implantation. We also showed that prolonged chondrogenic induction in vitro expedited endochondral ossification of cartilage discs in vivo.

The importance of ECM for chondrogenic differentiation of MSCs has been studied in vivo and in vitro. Developmental studies revealed interesting dynamics of ECM formation as the prechondrogenic mesenchyme was enriched with type I collagen whereas the mature cartilage was enriched with type II collagen [26, 27]. Although both collagen types have been used for in vitro chondrogenic induction of MSCs, a recent study found important differences between the early and late stage ECM during chondrogenesis [28–30]. Early-stage ECM was rich in type I collagen and other cell-binding proteins, and strongly induced chondrogenesis of MSCs [28–30]. In contrast, late-stage ECM was rich in type II collagen, deficient in type I collagen and other cell-binding proteins, and resulted in poor chondrogenic induction of MSCs [30]. Due to alternative splicing, mature chondrocytes synthesize type IIB procollagen that lacks a TGF-β-binding chordin-like domain [31]. Thus, it was proposed that type II collagen in ECM produced during late chondrogenesis disrupted TGF-β-mediated chondrogenesis [30].

Interestingly, we observed similar differences between the cartilage discs formed on membranes coated with type I collagen (Col1) versus type II collagen (Col2). Col1 discs exhibited better tissue properties as revealed by biochemical, histological, and mechanical analyses. The poor ECM deposition and mechanical properties of Col2 discs can be attributed to early deficits in the gene expression of cartilage markers. In agreement with the developmental studies, we found uniform expression of type I collagen within the first week of chondrogenic induction, which confirms its importance during early chondrogenesis of hMSCs.

Furthermore, Col1 coating enabled disc formation most reproducibly. In contrast, self-assembling hMSCs on uncoated membranes condensed and failed to form discs. Disc formation on type II collagen was less frequent than on type I collagen. Although we could not compare binding forces, our results suggest that type I collagen serves as a better anchor for the self-assembling hMSCs to resist condensation forces. Cell seeding density was shown to modulate cartilage formation by MSCs cultured in scaffold [32, 33]. Here, we showed that increasing the cell seeding density improved the compressive modulus, sGAG content, and thickness of the discs significantly. However, increasing the cell seeding density also promoted condensation and yielded less frequent disc formation. During mesenchymal condensation, MSCs undergo actomyosin contraction and cytoskeletal rearrangement [34]. It is likely that the increased condensation forces at high cell densities overcame the anchoring forces between the membrane ECM and the attached cells.

Disc culture formed hyaline cartilage while pellet culture formed fibrocartilage with more type I collagen. This outcome was attributed to the fibrogenic tensile forces associated with the increase in surface area of the pellet, but not the disc, which grew in thickness [8]. Disc culture also enhanced collagen network maturity [35]. We confirmed these findings and found that whereas pellet culture formed fibrocartilage with dense type I collagen at the surface, and disc culture formed hyaline cartilage with uniform deposition of sGAG and type II collagen. Furthermore, hMSCs differentiated in disc culture expressed more COL2A1 relative to ACAN than in pellet culture. Consequently, the discs exhibited a higher COL relative to sGAG content than the pellets. Morphologically, the discs were stratified and resembled the articular cartilage, with lubricin lining only the top surface.

As the primary goal of the overall study was to investigate a method for cartilage formation by hMSCs and its implications in vitro and in vivo, we chose to use a source of hMSCs that has been well characterized, shown to undergo robust differentiation, and extensively published. This enabled us to consistently generate cartilage tissues in different formats for in vitro and in vivo analyses and compare with previous work. However, the heterogeneity of MSCs is well documented and the findings from this study will need to be verified with cells isolated from different donors and sources such as the bone marrow, adipose tissue, and synovium. The lack of donor and cell source diversity is a limitation of this study that we seek to address in future investigations.

Although cartilage pellets and discs formed from self-assembling hMSCs both achieved structural integrity, other methods may be necessary to grow tissues for large-scale therapeutic applications. One such method for forming large and anatomically shaped cartilage is the fusion of pellets atop bone substrate that we demonstrated in our previous study [7]. Foreseeably, larger discs could also be formed by inducing cartilage formation from self-assembling hMSCs on a larger coated membrane. However, our studies suggest that improving the thickness and mechanical property of the discs comes at a cost of a lower yield of disc formation, necessitating the use of scaffolds for the cultivation of larger tissue constructs.

Depending on the extent of chondrogenic induction, pellets formed from MSCs are replaced by fibrous tissue in vivo or undergo endochondral ossification [10, 36]. To the best of our knowledge, the in vivo fate of the cartilage discs has not been studied. Thus, we assessed the in vivo implications of different culture regimens and found that both the pellets and the discs underwent endochondral ossification following prolonged chondrogenic induction, with remarkably different outcomes. Endochondral ossification of the pellets resembled callus maturation during long bone repair, whereby bone enveloped the pellet and cartilage loss progressed into the bulk of the tissue. Instead, endochondral ossification of the discs resembled epiphyseal cartilage maturation during skeletal development, whereby bone formed at the bottom and cartilage loss progressed toward the surface [37].

Recent studies showed that hypertrophic induction following chondrogenic induction promoted terminal chondrocyte differentiation from hMSCs in vitro and enhanced endochondral ossification in vivo. [11, 38]. Here, we showed that prolonged chondrogenic induction of self-assembling hMSCs in disc culture similarly expedited endochondral ossification in vivo. After 4 weeks of implantation, discs cultured for 10 weeks exhibited greater mineral volume and density than discs cultured for 6 weeks or 8 weeks. Mature bone and marrow stroma were present in the discs cultured for 10 weeks but not 6 weeks or 8 weeks, despite the same duration of implantation. Interestingly, discs cultured for 6 weeks mineralized at the rim whereas discs cultured for 10 weeks mineralized everywhere. This suggests early peripheral maturation that could be resulted from the normal compressive force at the insert wall [39]. These results demonstrate that the in vitro culture regimen can modulate endochondral ossification of cartilage formed by self-assembling hMSCs in vivo.

As the primary goal of the in vivo study was to evaluate cartilage stability, we only evaluated the tissue outcomes after 4 weeks of ectopic implantation. However, it is likely that the tissues would undergo further endochondral ossification with prolonged implantation. A recent 8-week implantation study on the fate of chondrocytes terminally differentiated from hMSCs found that chondrocytes released from lacunae during endochondral ossification underwent a reversion of differentiation to become marrow stromal cells within the ossicle [40]. With prolonged implantation, it is possible that terminally differentiated chondrocytes in both the discs and the pellets could have undergone a similar reversion, leading to the formation of bone and marrow stroma.

However, the presence of osteopontin and mineral indicated that during early stages of ectopic implantation, the in vivo environment promoted terminal differentiation of chondrocytes along a chondrogenic pathway. While a switch to osteogenic differentiation would also have resulted in the deposition of osteopontin and mineral, Movat’s pentachrome staining showed that the cells were residing in cartilage matrix, and were unlikely to have undergone a reversion of chondrogenic differentiation.

During in vitro culture, we did not observe a significant decrease in DNA content up to 6 weeks of chondrogenic differentiation in disc or pellet culture. This suggests that there was no progressive loss of cells during chondrogenic induction. Likewise, histological observations confirmed that the lacunae in the cartilage tissues remained well nucleated after implantation, suggesting the persistence of viable chondrocytes. However, prior studies suggest that terminal differentiation of chondrocytes can result in apoptosis, which mediates cartilage turnover [41].

The long-term in vivo stability of cartilage formed from MSCs remains a challenge [3, 42]. Recent studies found that modulation of Wnt signaling and hypoxia can enhance the in vivo stability of cartilage formed by MSCs in pellet culture [43, 44]. Conceivably, trophic and biophysical stimuli can improve the disc culture and enable the growth of a functional, organized and stable articular cartilage in vitro from MSCs. As the articular cartilage is anisotropic, implementation of spatiotemporal control during in vitro culture could recapitulate native gradients and further improve tissue organization and stability [45].

## Conclusions

The effects of culture parameters on cartilage disc formation by self-assembling hMSCs were evaluated in vitro and in vivo. Disc formation on a membrane could not be achieved without ECM coating. Type I collagen, uniformly expressed in the prechondrogenic mesenchyme, most frequently enabled disc formation and improved long-term tissue properties by enhancing early chondrogenesis. Increasing cell seeding density improved tissue properties, but also increased the condensation burden and resulted in less frequent disc formation. Disc culture formed stratified hyaline cartilage with uniform deposition of GAG and type II collagen, and pellet culture formed fibrocartilage callus with type I collagen at the surface. Following ectopic implantation, endochondral ossification of the discs and pellets exhibited different directionalities. Prolonged chondrogenic induction in vitro expedited endochondral ossification of the discs in vivo, starting at the rim. These results provide new directions for engineering cartilage and bone via a cartilage template from hMSCs by self-assembly methods.

## Abbreviations

COL: collagen
ECM: extracellular matrix
GAG: glycosaminoglycan
MSCs: mesenchymal stem cells
SCID: severe combined immunodeficiency
μCT: micro computed tomography
